# Clinical Inertia in SGLT2 Inhibitor Use Among Elderly Patients with Type 2 Diabetes and Chronic Kidney Disease: A Comparison of Regional and University Hospital Practice

**DOI:** 10.3390/geriatrics10060144

**Published:** 2025-11-06

**Authors:** Kyriaki Vafeidou, Ourania Psoma, Evangelos Apostolidis, Anastasia Sarvani, Michael Doumas, Kalliopi Kotsa, Vasileios Tsimihodimos, Theocharis Koufakis

**Affiliations:** 1 School of Medicine, Aristotle University of Thessaloniki, 54642 Thessaloniki, Greece; kiriakivfd@gmail.com (K.V.); vaggapo@gmail.com (E.A.); natasasar91@gmail.com (A.S.); doumasm@auth.gr (M.D.); kkalli@auth.gr (K.K.); 2 School of Medicine, University of Ioannina, 45110 Ioannina, Greece; raniapsoma@gmail.com (O.P.); vtsimi@uoi.gr (V.T.)

**Keywords:** SGLT2 inhibitors, chronic kidney disease, type 2 diabetes, elderly patients, clinical inertia

## Abstract

**Background/Objectives:** Type 2 diabetes (T2D) and chronic kidney disease (CKD) frequently coexist in older adults. Sodium–glucose cotransporter-2 inhibitors (SGLT2i) are recommended for renal and heart protection, yet their use in routine care remains inconsistent. We aimed to investigate differences in SGLT2i prescribing between regional and university hospital settings and assess whether such disparities persist after accounting for patient characteristics. **Methods:** In this retrospective analysis, patients were stratified by follow-up site (regional vs. university hospital). The primary outcome was SGLT2i use. Logistic regression models were adjusted for strong determinants of prescribing decisions, including age, sex, hypertension, dyslipidemia, heart failure, and estimated glomerular filtration rate. We tested the robustness of the results using additional analyses, including exclusion of frail patients and adjustment with propensity score methods, such as matching and inverse probability weighting (IPTW). **Results:** The study included 135 patients, of whom 80 were followed at the regional hospital and 55 at the university hospital. SGLT2i use was significantly lower in the regional setting (27.5% vs. 63.6%, *p* < 0.001). In adjusted models, university follow-up remained strongly associated with SGLT2i prescription [odds ratio 3.60, 95% confidence interval (CI) 1.61–8.03, *p* = 0.0018]. IPTW demonstrated 4.40-fold higher odds of SGLT2i use in the university hospital setting (95% CI 2.07–9.36, *p* < 0.001). **Conclusions:** These findings indicate that the lower use of SGLT2i among older adults with T2D and CKD followed in regional hospitals may reflect patterns consistent with clinical inertia, underscoring the importance of efforts to promote equitable and guideline-aligned prescribing practices across levels of care.

## 1. Introduction

Type 2 diabetes (T2D) is widely recognized as one of the defining public health problems of our time. Its impact extends well beyond the immediate consequences of hyperglycemia and includes an array of vascular and non-vascular complications [1]. Cardiovascular disease, kidney impairment, and other comorbidities accumulate with disease duration, contributing heavily to illness burden, reduced quality of life, and premature mortality [2]. The challenge of managing T2D is further heightened by the need for multiple medications and the progressive nature of the disease itself [3]. All these features reinforce the ongoing urgency of developing and implementing effective strategies of care.

The coexistence of T2D and chronic kidney disease (CKD) is especially worrisome. CKD often develops gradually, sometimes unnoticed, yet when combined with diabetes the two conditions interact in a way that accelerates deterioration on both sides [4]. Patients living with both T2D and CKD face substantially higher risks of hospitalization, cardiovascular complications, and early death [5]. At the same time, declining renal function restricts therapeutic options. Although new pharmacological tools—most notably sodium-glucose cotransporter-2 inhibitors (SGLT2i)—have created opportunities for both renal and cardiovascular protection, their adoption in everyday practice has been uneven and far from optimal [6].

Older adults with T2D bring additional challenges. Frailty, cognitive decline, and limitations in daily functioning are common, and they complicate treatment decisions [7]. In those with CKD, the risk of hypoglycemia, polypharmacy, and drug–drug interactions become even more pressing [8]. This is also the group that might benefit the most from therapies such as SGLT2i, given their ability to protect both kidney and heart [9].

Care pathways also vary according to setting. In most healthcare systems, patients with T2D are not only managed in tertiary or university hospitals but also in smaller district hospitals and primary care facilities. These environments differ in their access to specialists, in the speed with which updated guidelines are adopted, and in prescribing behavior [10]. Such differences create fertile ground for what has been termed “clinical inertia.” This refers to the failure to initiate or escalate treatment when evidence clearly supports doing so. It is a multifactorial issue, shaped by healthcare structures, physician perceptions, and patient-related barriers [11]. Its consequences are substantial. Delays in evidence-based treatment often translate into avoidable complications and poorer long-term outcomes [12].

Against this background, the present analysis set out to examine prescribing practices for SGLT2i among elderly patients with T2D and CKD who were managed either in a regional hospital or in a university-affiliated hospital. The aim was twofold: first, to investigate whether there are measurable differences in uptake of SGLT2i between these care settings; and second, to explore whether such disparities can be attributed to differences in patient characteristics. In doing so, the study seeks to clarify to what extent clinical inertia may be shaping real-world therapeutic decisions.

## 2. Methods

### 2.1. Study Design

This analysis was conducted as part of a previously published retrospective observational cohort study of elderly patients with T2D and CKD [13]. For clarity, the main eligibility criteria are restated here in condensed form. Patients included were adults aged 65 years and older with both T2D and established CKD, defined by reduced estimated glomerular filtration rate (eGFR < 60 mL/min/1.73 m^2^) and/or elevated urine albumin-to-creatinine ratio (UACR > 30 mg/g) [14]. Exclusion criteria included type 1 diabetes, acute kidney injury, incomplete clinical data, and any contraindication to SGLT2i use (e.g., eGFR < 20 mL/min/1.73 m^2^ or severe disorders of the urinary system). Patients with a documented history of recurrent or complicated urinary tract infections (UTIs) during previous SGLT2i therapy were excluded, as were those with indwelling urinary catheters or receiving antibiotic treatment for active UTI at the index visit. These exclusions were applied to minimize bias related to drug tolerability or safety considerations. Because this was a cross-sectional analysis, incident UTIs after baseline were not assessed. Participants were recruited between November 2024 and February 2025. For each eligible participant, detailed information on laboratory parameters, comorbidities, and medications was extracted from the hospital’s electronic medical records.

To preserve anonymity and avoid potential stigmatization, the specific names of participating hospitals are not reported in this analysis. Instead, hospitals are described in generic terms as a “regional hospital” and a “university hospital.” Patients were stratified according to the type of center providing follow-up. The primary outcome was the proportion of patients prescribed an SGLT2i. Secondary analyses examined between-site differences in demographic, clinical, and biochemical characteristics, and evaluated whether the observed site effect on SGLT2i use persisted after adjustment for key covariates.

### 2.2. Statistical Analysis

Descriptive statistics were used to summarize demographic and clinical characteristics. Continuous variables were expressed as medians with interquartile ranges and compared using the Mann–Whitney U test, while categorical variables were expressed as counts and percentages and compared using chi-square tests. To account for potential confounding, we constructed multivariable logistic regression models with SGLT2i use as the dependent variable and site (regional vs. university) as the main predictor. The models adjusted for strong determinants of prescribing decisions, including age, sex, hypertension, dyslipidemia, presence of heart failure (HF), and eGFR. UACR was too sparsely available to be included in the main models. Although the prevalence of dementia differed between groups, it was not included as a covariate in multivariable models because of its low overall prevalence, limited direct link to prescribing according to current guidelines, and concerns about model overfitting. Instead, frailty and acute-illness exclusions [e.g., age ≥ 90 years, hemoglobin < 10 g/dL, or C-reactive protein (CRP) > 10 mg/dL] were performed as sensitivity analyses to indirectly account for vulnerability related to cognitive and functional status.

In addition, a propensity score (PS) analysis was undertaken. PS were derived from a logistic model predicting the likelihood of university follow-up given baseline covariates (age, sex, hypertension, dyslipidemia, HF, eGFR). Patients were matched 1:1 between sites using nearest-neighbor matching within a caliper of 0.2 standard deviations of the logit PS. As a complementary approach, inverse probability weighting (IPTW) with stabilized weights was applied, trimming extreme weights at the 99th percentile. IPTW uses the propensity score to assign weights to each patient, creating a pseudo-population in which baseline covariates are more evenly distributed between groups, thereby mimicking the balance of a randomized trial. Standardized mean differences were used to assess balance before and after matching or weighting. The number of variables included in multivariable models was restricted to maintain an adequate events-per-variable ratio and to minimize the risk of model overfitting. This approach was complemented by PS–based methods (matching and IPTW) to further mitigate residual confounding. All analyses were performed with two-tailed tests, and a *p*-value < 0.05 was considered statistically significant. Statistical analyses were conducted using R software (version 4.3.1; R Foundation for Statistical Computing, Vienna, Austria).

### 2.3. Ethical Approval

The study received approval from the Bioethics Committee of the Aristotle University of Thessaloniki (approval number: 1/date: 12 November 2024). Informed consent was obtained from all subjects involved in the study. All data were anonymized prior to analysis, and the study was conducted in line with the principles of the Declaration of Helsinki.

## 3. Results

### 3.1. Demographics and Baseline Characteristics

The study included 135 patients, of whom 80 were followed at the regional hospital and 55 at the university hospital. The median age was slightly higher in the regional cohort [83 (77–86) vs. 79 (76–84) years], although this difference did not reach statistical significance (*p* = 0.058). Dementia was significantly more prevalent in the regional group (16.2% vs. 1.8%, *p* = 0.016). By contrast, cardiometabolic comorbidities were more frequently documented in the university hospital cohort, with higher rates of dyslipidemia (80.0% vs. 43.8%, *p* < 0.001) and hypertension (89.1% vs. 70.0%, *p* = 0.016), as well as greater use of concomitant therapies such as glucagon-like peptide-1 receptor agonists (GLP-1 RA) (36.4% vs. 7.5%, *p* < 0.001) and renin–angiotensin–aldosterone system (RAAS) inhibitors (81.8% vs. 61.3%, *p* = 0.018).

Hemoglobin was significantly lower and CRP significantly higher in the regional cohort (both *p* < 0.05), consistent with a more frail or inflammatory profile. HF prevalence and median eGFR did not differ significantly between groups. These between-group differences are not unexpected, as university hospitals typically manage patients with more complex cardiometabolic disease. UACR values were available for a limited subset of patients and did not differ significantly between those treated and not treated with SGLT2i (101.00 ± 133.56 vs. 103.50 ± 139.30 mg/g, *p* = 0.769). Given the small sample size, these data should be interpreted with caution. Table 1 presents the demographic characteristics, key laboratory values, and comorbidities of the study population according to hospital care group.

### 3.2. Comparison of SGLT2i Prescribing

SGLT2i use differed markedly by site. Only 27.5% of regional hospital patients received an SGLT2i compared with 63.6% at the university hospital (*p* < 0.001). This difference persisted after multivariable adjustment: in the main logistic regression, university hospital follow-up was associated with 3.6-fold higher odds of SGLT2i prescription [odds ratio (OR) 3.60, 95% confidence interval (CI) 1.61–8.03; *p* = 0.0018]. Although dyslipidemia and hypertension were more common in the university hospital cohort, they did not emerge as significant predictors of SGLT2i use in either univariable or multivariable analyses (Table 2). When UACR values were included in the regression model for the subset of patients with available data (*n* = 13), the direction and significance of the association between care setting and SGLT2i prescribing remained unchanged.

Several additional analyses were conducted to explore the robustness of the findings. When patients considered frail or acutely unwell—defined as those aged 90 years or older, with hemoglobin levels below 10 g/dL, or with CRP values greater than 10 mg/dL—were excluded, the disparity between sites persisted (OR 4.89, 95% CI 1.61–14.83; *p* = 0.005). No significant interactions were observed between site and either age or eGFR. Finally, analyses based on PS provided consistent evidence of differences: matching resulted in an absolute risk difference of +21.2 percentage points (95% CI 0.0–42.4), while IPTW demonstrated a more pronounced association (OR 4.40, 95% CI 2.07–9.36; *p* < 0.001) (Table 3).

## 4. Discussion

This analysis provides an exploratory assessment of prescribing patterns among elderly patients with T2D and CKD. A noticeably lower use of SGLT2i was observed among patients followed in a regional hospital compared with those managed in a university-affiliated center. The difference remained significant after adjustment for major demographic and clinical variables and was robust across sensitivity analyses, including models excluding frail participants and those using PS methods. Taken together, these findings suggest a consistent and reproducible difference in prescribing behavior across care settings, rather than implying causality. While international guidelines strongly recommend the use of SGLT2i in this population [15], translation into routine practice may vary between healthcare environments, reflecting possible system-level, educational, or structural influences that merit further exploration.

Although the present findings arise from the Greek healthcare system, their implications likely extend beyond this specific country. Structural differences between tertiary centers and smaller regional hospitals or primary care settings are common internationally, and the variable adoption of novel therapies is a global concern rather than a local peculiarity [16]. Against this background, regionally focused analyses acquire particular value. Large clinical trials provide essential evidence, but the translation of their results into everyday practice depends heavily on local resources [17,18]. The present study helps to expose gaps that may be obscured by national averages or international surveys and emphasizes that inequities in therapy persist even within a single health system, underlining the need for further investigation and targeted interventions.

The reasons behind the observed discrepancy are likely multifactorial. System-level barriers may play a role: regional hospitals often have more limited access to diabetology or nephrology specialists, fewer structured educational programs, and less exposure to the latest guideline updates [19]. Physician-related factors cannot be dismissed either. Prescribers may be less familiar with newer therapies, or hesitant to use them in frail elderly patients due to concerns about tolerability or side effects [20]. From the patient perspective, issues such as limited health literacy, competing comorbidities, and cost considerations may also influence prescribing decisions [21]. None of these explanations are mutually exclusive, and the interaction between them is complex. In the Greek healthcare system, both primary care physicians and specialists are equally authorized to prescribe novel antidiabetic agents, including SGLT2i and GLP-1 RA, through the national electronic prescription system. Therefore, differences in formal prescribing rights or reimbursement procedures are unlikely to explain the observed variation. Nevertheless, institutional familiarity with electronic approval systems, local administrative routines, or specialist consultation pathways may still influence uptake.

When viewed against the background of existing research, the pattern is not entirely surprising. Several studies across Europe and North America have documented gaps between guideline recommendations and real-world practice in the use of SGLT2i [22,23]. Reports often highlight higher uptake in academic or specialized centers compared to community practices [24]. It should also be noted that the observed gap in prescribing is not confined to SGLT2i. In particular, use of GLP-1 RA and RAAS inhibitors was markedly lower in the regional hospital cohort, despite well-documented cardiovascular and renal benefits of these agents. These findings suggest that clinical inertia extends beyond a single drug class and reflects a more general hesitancy in implementing modern, evidence-based pharmacotherapy and translating evidence from cardiovascular outcome trials into routine prescribing [25].

Clinical inertia in primary or secondary care is a well-described phenomenon. Previous work has suggested that between 30% and 50% of patients with poorly controlled T2D may remain on unchanged therapy for prolonged periods, sometimes years, even when intensification is clinically indicated [26]. For CKD, delays in initiating renoprotective therapies such as RAAS inhibitors have also been described, particularly in non-specialist settings [27]. The present findings therefore align with a broader body of evidence: inertia is common, and it may be more pronounced in settings where specialist input is less readily available.

One avenue to address clinical inertia lies in strengthening the education of primary care physicians. Studies have demonstrated that targeted educational interventions, including structured training programs and guideline dissemination workshops, can significantly improve prescribing of evidence-based therapies in T2D and CKD [28]. For example, programs incorporating case-based learning and feedback loops have been shown to increase initiation rates of novel therapies in community practices [29]. While education alone is unlikely to resolve all barriers, it represents a crucial element in bridging the gap between evidence and practice. Additional measures that may support better alignment of primary care physicians with clinical recommendations include reducing workload pressures and extending the length of consultations, as both have been identified as major obstacles to consistent guideline adherence [30].

Several strengths of this work should be acknowledged. The study was based on detailed clinical data, enabling adjustment for relevant demographic and clinical covariates, including age, comorbidities, HF, and kidney function. Multiple analytic strategies were applied—ranging from conventional regression to PS techniques—enhancing the robustness of findings. Importantly, the focus on elderly patients with both T2D and CKD addresses a group often underrepresented in clinical trials, yet highly relevant in real-world practice.

Nonetheless, the findings should be interpreted in light of important limitations. The analysis was restricted to a modest sample size, which may reduce statistical power for some subgroup comparisons and interaction tests. UACR data were sparsely available, precluding full adjustment for albuminuria. Residual confounding due to unmeasured factors such as diabetes duration, glycemic control (HbA1c), socioeconomic status, or physician-level characteristics cannot be excluded. Although multiple strategies, including PS adjustment and sensitivity analyses, were used to minimize bias, causal inference is not possible within this cross-sectional framework. The regression models were intentionally parsimonious to preserve an adequate events-per-variable ratio and avoid overfitting, and the observed effect sizes should be interpreted with appropriate caution. These estimates likely reflect meaningful differences in prescribing behavior between settings rather than causality, but some degree of overestimation due to unmeasured confounding cannot be entirely ruled out. Furthermore, while anonymization was necessary to avoid stigmatization, it also limits the ability to contextualize results within specific local health system structures. Detailed echocardiographic parameters were not consistently available, and heart failure was therefore recorded as a binary variable. This precluded stratification by ejection fraction, which could provide additional insights into prescribing patterns. Future research incorporating standardized echocardiographic data could address this question. The analysis did not incorporate socioeconomic data. However, in the context of the Greek National Health System, access to novel antidiabetic agents is provided at little or no cost, even for individuals without social insurance. As such, the influence of socioeconomic barriers on prescribing is likely to be minimal in this setting. Although prescribing rights for SGLT2i and GLP-1 RA are uniform across care levels within the Greek healthcare system, site-level differences in administrative workflow or clinical culture could still contribute to the observed variability. Frailty was approximated using pragmatic laboratory and age-based criteria rather than validated functional scales, which may not fully reflect functional vulnerability. Finally, as this was a cross-sectional exploratory analysis, the study did not assess downstream outcomes such as kidney function decline, cardiovascular events, or mortality. Future longitudinal research is warranted to determine whether the observed differences in prescribing translate into measurable variations in clinical outcomes. In this context, the interpretation of our findings should remain exploratory. Multiple explanations—ranging from patient complexity to institutional prescribing culture and access to specialist input—may account for the observed patterns. Qualitative investigations and longitudinal designs will be required to confirm these associations and to understand their drivers.

## 5. Conclusions

In conclusion, this study identified a clear disparity in the prescription of SGLT2i between patients followed in regional hospitals and those managed in university hospital settings. The difference persisted after adjustment for demographic and clinical variables, pointing to practice patterns rather than patient profiles as the main driver. Moreover, the underuse extended beyond SGLT2i to other modern cardiometabolic therapies, suggesting a broader pattern of clinical inertia. Although the analysis was conducted in a Greek healthcare context, the observations mirror reports from other countries where adoption of newer, evidence-based treatments remains uneven. This highlights the need to recognize that barriers are not solely clinical but are also shaped by institutional culture, specialist access, and continuing education opportunities.

Future initiatives should focus on actionable strategies. Targeted training programs for primary care physicians—who provide much of the long-term management for patients with T2D and CKD—have already shown promise in improving uptake of the new renoprotective therapies. Strengthening the links between community care and tertiary centers, while ensuring appropriate referral paths, could also narrow these gaps. Addressing therapeutic inertia will therefore require coordinated interventions across system, provider, and patient levels. The ultimate goal is to ensure that patients can fully benefit from therapies shown to mitigate the increased cardiorenal risk resulting from the combined burden of T2D, CKD and advanced age.

## Figures and Tables

**Table 1:** Demographics and comorbidities by hospital type.

Variable	Regional Hospital (*n* = 80)	University Hospital (*n* = 55)	*p*-Value
Age, years (median [IQR])	83 (77–86)	79 (76–84)	0.058
Female sex, %	48.8%	52.7%	0.66
Hypertension, %	70.0%	89.1%	0.016
Dyslipidemia, %	43.8%	80.0%	<0.001
Heart failure, %	28.7%	27.3%	1.00
Dementia, %	16.2%	1.8%	0.016
Hemoglobin, g/dL	10.8 (9.1–11.9)	12.7 (11.2–13.8)	<0.001
CRP, mg/dL	4.2 (1.2–10.5)	1.0 (0.5–3.2)	0.023
eGFR, mL/min/1.73 m^2^	35.5 (30.8–47.2)	39.0 (31.0–50.0)	0.208

CRP: C-reactive protein; eGFR: Estimated glomerular filtration rate; IQR: interquartile range.

**Table 2:** Logistic regression models for predictors of SGLT2i use.

Predictor	Odds Ratio [95% CI]	*p*-Value
University vs. Regional site	3.60 (1.61–8.03)	0.0018
Age (per year)	0.93 (0.87–0.99)	0.017
Female sex	1.12 (0.51–2.49)	0.78
Hypertension	1.45 (0.62–3.42)	0.39
Dyslipidemia	1.38 (0.61–3.12)	0.44
Heart failure	2.05 (0.82–5.13)	0.12
eGFR (per mL/min/1.73 m^2^)	0.95 (0.91–0.99)	0.011

CI: confidence interval; eGFR: Estimated glomerular filtration rate; SGLT2i: sodium-glucose cotransporter-2 inhibitors.

**Table 3:** Propensity score analyses of SGLT2i use.

Analysis Type	Effect Estimate	95% CI	*p*-Value
PS-matched pairs	+21.2% absolute difference	0.0–42.4	0.118
IPTW (weighted logit)	OR 4.40	2.07–9.36	<0.001

CI: confidence interval; IPTW: inverse probability of treatment weighting; OR: odds ratio; PS: propensity score; SGLT2i: sodium-glucose cotransporter-2 inhibitors.

## Data Availability

The data presented in the study are available on request from the corresponding author. The data are not publicly available due to privacy restrictions of the Greek National Health System.
